# NOXclass: prediction of protein-protein interaction types

**DOI:** 10.1186/1471-2105-7-27

**Published:** 2006-01-19

**Authors:** Hongbo Zhu, Francisco S Domingues, lngolf Sommer, Thomas Lengauer

**Affiliations:** 1 Max-Planck-lnstitut für Informatik, Stuhlsatzenhausweg 85, 66123 Saarbrücken, Germany

## Abstract

**Background:**

Structural models determined by X-ray crystallography play a central role in understanding protein-protein interactions at the molecular level. Interpretation of these models requires the distinction between non-specific crystal packing contacts and biologically relevant interactions. This has been investigated previously and classification approaches have been proposed. However, less attention has been devoted to distinguishing different types of biological interactions. These interactions are classified as obligate and non-obligate according to the effect of the complex formation on the stability of the protomers. So far no automatic classification methods for distinguishing obligate, non-obligate and crystal packing interactions have been made available.

**Results:**

Six interface properties have been investigated on a dataset of 243 protein interactions. The six properties have been combined using a support vector machine algorithm, resulting in NOXclass, a classifier for distinguishing obligate, non-obligate and crystal packing interactions. We achieve an accuracy of 91.8% for the classification of these three types of interactions using a leave-one-out cross-validation procedure.

**Conclusion:**

NOXclass allows the interpretation and analysis of protein quaternary structures. In particular, it generates testable hypotheses regarding the nature of protein-protein interactions, when experimental results are not available. We expect this server will benefit the users of protein structural models, as well as protein crystallographers and NMR spectroscopists. A web server based on the method and the datasets used in this study are available at .

## Background

Protein-protein interactions play important roles in many biological processes. Structural models of the complexes resulting from these interactions are necessary to understand those processes at the molecular level. Among the different techniques which can be employed to determine the structures of protein complexes, X-ray crystallography is still the most popular [1]. However, not all interactions observed in structures of protein complexes determined by X-ray crystallography are biologically relevant. Many of them are formed during the crystallization process and would not appear *in vivo*. Such crystal packing contacts are non-specific and have no biological function associated [2]. The determination of the quaternary structure of protein complexes remains a field of active research [2-9].

In addition, there are diverse types of biological interactions [10]. Protomers from obligate complexes do not exist as stable structures *in vivo*, whereas protomers of non-obligate complexes may dissociate from each other and stay as stable and functional units. Similarly, protein complexes have been divided as permanent or transient according to their lifetime.

A number of studies have examined properties of protein-protein interfaces in order to discriminate biologically relevant interactions and non-biological interactions resulting from crystal packing contacts. It has been shown that biological interactions tend to have larger interface size than non-biological interactions [2-6,11]. PQS [5], which uses interface size as its main discriminant, separated true from false homodimers with an accuracy of 78% on a non-redundant dataset [12]. A 400 Å^2 ^cutoff for interface size between biological interactions and non-biological interactions is used by PQS. Ponstingl and coworkers reported an optimal cutoff of 856 Å^2 ^for differentiating homodimers and monomers [6]. However, counterexamples were also observed for which this criterion failed [4,6]. Amino acid composition of the interface is another well-analyzed property for identifying biological interactions [3,9,13,14]. It has been reported that the amino acid composition of biological interfaces is different from that of the rest of protein surface [9,13,14]. On the other hand, Carugo and collaborators showed that the chemical composition of crystal packing contacts is very similar to that of the rest of the surface as a whole [3]. The importance of residue conservation in the identification of the oligomeric state of protein complexes has been investigated. Using a neural network algorithm for combining the size and conservation measures of the interface, biological homodimeric interactions and crystal packing contacts can be successfully classified with an accuracy of 98.3% [12]. Zhang *et al*. introduced statistical learning methods to predict protein quaternary structures based on protein sequence information [15].

Similar properties have been employed for identifying protein-protein interaction sites. Jones and Thornton analyzed six physicochemical interface properties and used them for predicting interaction sites [13,16]. Gallet *et al*. identified residues involved in protein interaction sites based on hydrophobicity [17]. Zhou and Shan used sequence profiles of neighboring residues and solvent accessibility of a target residue [18]. Also, residue conservation has been employed to infer functional hot spots at the protein surface [19-22]. The approaches are based on the assumption that key residues involved in biologically relevant interactions are more strongly conserved in evolution than the rest of protein surfaces. Though several conservation scores have proven useful, there is still room for improvement [23]. Different properties have been combined with a support vector machine (SVM) implementation in order to predict protein-protein binding sites [24,25]. Some efforts have been made to discriminate different types of biological interactions. Transient protein-protein interactions, including both homodimers and heterodimers, have been characterized at the structural level [26]. This work revealed that interfaces of transient complexes have smaller area, and are more planar and polar on average than those of stable homodimers. In addition, interface residues of transient homodimers have been found to be more conserved than the other surface residues. Gunasekaran and coworkers reported that both per-residue surface area and interface area of ordered proteins (involving non-obligate interactions) are much smaller than those of disordered proteins (involving obligate interactions) [27]. Recently, De *et al*. performed a statistical analysis of the interface properties for obligate and non-obligate interactions [28]. They reported that obligate interfaces have more contacts than non-obligate interfaces. And these contacts are mainly nonpolar. Involvement of secondary structure elements at interfaces were reported to be significantly different. In a recent paper, Mintseris and Weng investigated the difference between obligate and transient complexes from an evolutionary point of view [29]. In obligate interactions, interface residues were reported to be significantly more conserved than those in transient interactions. In addition, the coevolution rate was observed to be lower for obligate interaction partners than for transient interaction partners. In general, obligate and non-obligate proteins have been shown to have distinct interaction preferences. Nevertheless, there is no single interface property with a clear cutoff on whose basis one can discriminate between the different protein interaction types. This is not surprising given the complexity and diversity of protein interactions. Mintseris and Weng used atomic contact vectors to discriminate obligate from non-obligate interactions [30]. They achieved respectable accuracy (91%) in such a classification problem. Clearly, there has been considerable progress in the analysis and classification of the different types of interactions, but so far no method has been made available for the prediction of protein-protein interaction types.

In this paper, first we investigate six interface properties for a set of non-redundant protein-protein interactions. These properties are interface area, ratio of interface area to protein surface area, amino acid composition of the interface, correlation between amino acid compositions of interface and protein surface, interface shape complementarity, and conservation of the interface. Then we trained an SVM classifier with these interface properties to differentiate not only biological interaction from crystal packing contacts, but also obligate interactions from non-obligate interactions. We constructed a two-stage SVM to handle the three-class classification problem. Our SVM classifier achieved an accuracy of 91.8% using leave-one-out cross-validation on the non-redundant dataset containing 243 interactions.

## Methods

### Training data

We compiled a non-redundant data set with three types of protein-protein interactions from several sources. Here, every interaction involves two protomers, which refer to the two polypeptide chains in the protein complex. There may be more than two protomers per complex, resulting in several interactions. When considering a protein-protein interaction, only the two protomers involved are relevant.

Obligate interactions were taken from a previously compiled set [25]. Non-obligate interactions were obtained from both a set of non-obligate interactions [25] and a set of transient interactions [31], which are non-obligate by definition. To remove redundancies [32], these interactions were first divided into groups. Each group is defined by the two SCOP families to which the two interaction protomers belong. Then we selected within each group the interaction whose complex has the highest AEROSPACI score [33]. The AEROSPACI score is a measure of the quality of the structural models available in the Protein Data Bank (PDB) [34]. After removing redundancy, we have 94 obligate interactions and 88 non-obligate interactions. Some problematic cases were found and removed from the set. For example, small ligands were found in some interfaces, or there was an interaction between two different parts of the same protein that was cleaved into two chains as a result of proteolysis. In total we removed eight cases from the obligate set (1bbh, 1bft, 1g4y, 1mka, 1nsy, 1scf, 1vfr and 5hvp) and six entries from the non-obligate set (1bpl, 1noc, 1fap, 1bmq 1ef1 and 2kau). The ConSurf server [21] was used to derive the conservation scores for these protein sequences. Only for a subset of these interactions we could obtain conservation scores for the protomers involved. In this subset of interactions, there are 75 obligate interactions and 62 non-obligate interactions. Enzyme homodimers predominate in the obligate set, but the set also includes other types of proteins, like transcription regulators or membrane receptors. The non-obligate set includes many interactions between enzyme and inhibitors, but it also includes other types of interactions like different examples of receptor-ligand interactions or transient signaling complexes.

A set of crystal packing contacts was compiled from the PDB in two steps. First we collected a non-redundant set of biological dimers from the PDB. We selected all dimeric complexes as defined in the PDB file sections REMARK 300 and REMARK 350. A similar procedure as described above was used to eliminate the redundancy in the set. The dimers were grouped according to the pair of SCOP families to which they belong. For each group the complexes with AEROSPACI scores below 0.5 were removed. The biological units for the remaining dimers were confirmed by manually inspecting the relevant literature. Then, for each group the dimer with the highest AEROSPACI score was selected. In total we collected 120 dimers. Second, for the selected 120 dimers we rebuilt unit cells and chose the largest non-biological interface in each unit cell for our final set of crystal packing contacts. We obtained 120 crystal packing contacts with this procedure, but for only 106 of them we could obtain conservation scores. In total, we gathered 243 protein-protein interactions of which 75 are obligate interactions, 62 are non-obligate interactions and 106 are crystal packing contacts. We will refer to this final dataset as BNCP-CS. The PDB ids are listed in Table 1.

### Definition of interface properties

In order to characterize the different types of protein-protein interactions, we analyzed the following six interface properties: interface area, ratio of interface area to protein surface area, amino acid composition of the interface, correlation between amino acid compositions of interface and protein surface, gap volume index, and conservation score of the interface. A residue is defined as being part of the interface if its solvent accessible surface area (SASA) decreases by > 1 Å^2 ^upon the formation of the complex [13]. A protein-protein interface is defined to be the ensemble of all interface residues from both protomers. Solvent accessible surface areas for residues were calculated using NACCESS [35], with a probe sphere of radius 1.4 Å.

#### Interface area

Interface area is defined as one half of the total decrease of SASA (ΔSASA) of the two protomers upon the formation of the interaction:

Interface Area=12(SASAa+SASAb−SASAab)
 MathType@MTEF@5@5@+=feaafiart1ev1aaatCvAUfKttLearuWrP9MDH5MBPbIqV92AaeXatLxBI9gBaebbnrfifHhDYfgasaacH8akY=wiFfYdH8Gipec8Eeeu0xXdbba9frFj0=OqFfea0dXdd9vqai=hGuQ8kuc9pgc9s8qqaq=dirpe0xb9q8qiLsFr0=vr0=vr0dc8meaabaqaciaacaGaaeqabaqabeGadaaakeaacqqGjbqscqqGUbGBcqqG0baDcqqGLbqzcqqGYbGCcqqGMbGzcqqGHbqycqqGJbWycqqGLbqzcqqGGaaicqqGbbqqcqqGYbGCcqqGLbqzcqqGHbqyiiaacqWF9aqpdaWcaaqaaiabigdaXaqaaiabikdaYaaacqGGOaakcqqGtbWucqqGbbqqcqqGtbWucqqGbbqqdaWgaaWcbaGaemyyaegabeaakiabgUcaRiabbofatjabbgeabjabbofatjabbgeabnaaBaaaleaacqWGIbGyaeqaaOGaeyOeI0Iaee4uamLaeeyqaeKaee4uamLaeeyqae0aaSbaaSqaaiabdggaHjabdkgaIbqabaGccqGGPaqkaaa@57F4@

where *a *and *b *are two protomers in the complex *ab*; SASA_*a*_, SASA_*b *_and SASA_*ab *_are the SASA values for *a*, *b*, and *ab*, respectively. The native complex may contain additional protomers, but they are not considered.

#### Interface area ratio

Biological interactions that involve a small protomer cannot have large interface areas. This applies to some enzyme-inhibitor complexes, for instance. Therefore, we defined a new feature, in which the interface area is normalized by the SASA of the smaller protomer in the complex:

Interface Area Ratio=Interface Areamin⁡(SASAa,SASAb)
 MathType@MTEF@5@5@+=feaafiart1ev1aaatCvAUfKttLearuWrP9MDH5MBPbIqV92AaeXatLxBI9gBaebbnrfifHhDYfgasaacH8akY=wiFfYdH8Gipec8Eeeu0xXdbba9frFj0=OqFfea0dXdd9vqai=hGuQ8kuc9pgc9s8qqaq=dirpe0xb9q8qiLsFr0=vr0=vr0dc8meaabaqaciaacaGaaeqabaqabeGadaaakeaacqqGjbqscqqGUbGBcqqG0baDcqqGLbqzcqqGYbGCcqqGMbGzcqqGHbqycqqGJbWycqqGLbqzcqqGGaaicqqGbbqqcqqGYbGCcqqGLbqzcqqGHbqycqqGGaaicqqGsbGucqqGHbqycqqG0baDcqqGPbqAcqqGVbWBcqGH9aqpdaWcaaqaaiabbMeajjabb6gaUjabbsha0jabbwgaLjabbkhaYjabbAgaMjabbggaHjabbogaJjabbwgaLjabbccaGiabbgeabjabbkhaYjabbwgaLjabbggaHbqaaiGbc2gaTjabcMgaPjabc6gaUjabcIcaOiabbofatjabbgeabjabbofatjabbgeabnaaBaaaleaacqWGHbqyaeqaaOGaeiilaWIaee4uamLaeeyqaeKaee4uamLaeeyqae0aaSbaaSqaaiabdkgaIbqabaGccqGGPaqkaaaaaa@6B26@

where SASA_*a *_and SASA_*b *_are the SASA values for protomers *a *and *b*, respectively.

#### Amino acid composition of the interface

We calculated both number-based and area-based amino acid composition [9]. The number-based amino acid composition (*v*_*n*_) is defined as the frequency of each type of the 20 standard amino acids in the protein-protein interface. By weighting each residue with its ΔSASA, the area-based amino acid composition *v*_*a *_is computed:

va,i=1…20=12 Interface Area∑r,type(r)=iΔSASA(r)
 MathType@MTEF@5@5@+=feaafiart1ev1aaatCvAUfKttLearuWrP9MDH5MBPbIqV92AaeXatLxBI9gBaebbnrfifHhDYfgasaacH8akY=wiFfYdH8Gipec8Eeeu0xXdbba9frFj0=OqFfea0dXdd9vqai=hGuQ8kuc9pgc9s8qqaq=dirpe0xb9q8qiLsFr0=vr0=vr0dc8meaabaqaciaacaGaaeqabaqabeGadaaakeaacqWG2bGDdaWgaaWcbaGaemyyaeMaeiilaWIaemyAaKMaeyypa0JaeGymaeJaeSOjGSKaeGOmaiJaeGimaadabeaakiabg2da9maalaaabaGaeGymaedabaGaeGOmaiJaaGPaVlabbMeajjabb6gaUjabbsha0jabbwgaLjabbkhaYjabbAgaMjabbggaHjabbogaJjabbwgaLjabbccaGiabbgeabjabbkhaYjabbwgaLjabbggaHbaadaaeqbqaaiabgs5aejabbofatjabbgeabjabbofatjabbgeabjabcIcaOiabdkhaYjabcMcaPaWcbaGaemOCaiNaeeilaWIaeeiDaqNaeeyEaKNaeeiCaaNaeeyzauMaeiikaGIaemOCaiNaeiykaKIaeyypa0JaemyAaKgabeqdcqGHris5aaaa@658F@

where type(*r*) is the type of the amino acid of residue *r*.

The Δ*ν *distance between two vectors *ν *and *ν' *of amino acid composition, number or area-based, is defined as [9,14]:

(Δv)2=119∑i=120(vi−vi')2
 MathType@MTEF@5@5@+=feaafiart1ev1aaatCvAUfKttLearuWrP9MDH5MBPbIqV92AaeXatLxBI9gBaebbnrfifHhDYfgasaacH8akY=wiFfYdH8Gipec8Eeeu0xXdbba9frFj0=OqFfea0dXdd9vqai=hGuQ8kuc9pgc9s8qqaq=dirpe0xb9q8qiLsFr0=vr0=vr0dc8meaabaqaciaacaGaaeqabaqabeGadaaakeaacqGGOaakcqGHuoarcqWG2bGDcqGGPaqkdaahaaWcbeqaaiabikdaYaaakiabg2da9maalaaabaGaeGymaedabaGaeGymaeJaeGyoaKdaamaaqahabaGaeiikaGIaemODay3aaSbaaSqaaiabdMgaPbqabaGccqGHsislcqWG2bGDdaqhaaWcbaGaemyAaKgabaGaei4jaCcaaOGaeiykaKYaaWbaaSqabeaacqaIYaGmaaaabaGaemyAaKMaeyypa0JaeGymaedabaGaeGOmaiJaeGimaadaniabggHiLdaaaa@4862@

#### Correlation between amino acid compositions of interface and protein surface

The amino acid composition of the biological interface was shown to be significantly different from that of the rest of the protein surface [36]. It is reasonable to expect the amino acid composition of the crystal packing interface to be similar to that of the rest of the protein surface. To measure this effect, the Pearson's correlation coefficients between the amino acid compositions of interface and surface were calculated. These correlations were calculated for both number-based and area-based amino acid compositions.

#### Gap volume index

It has been shown that the protein-protein interfaces are more complementary in obligate complexes than those in non-obligate complexes [9,37]. The gap volume index is one of the measurements for interface complementarity [9]. Since gap volume is dependent on protein size, this feature is computed by normalizing the gap volume between protomers with their interface area:

Gap Volume Index=Gap VolumeInterface Area
 MathType@MTEF@5@5@+=feaafiart1ev1aaatCvAUfKttLearuWrP9MDH5MBPbIqV92AaeXatLxBI9gBaebbnrfifHhDYfgasaacH8akY=wiFfYdH8Gipec8Eeeu0xXdbba9frFj0=OqFfea0dXdd9vqai=hGuQ8kuc9pgc9s8qqaq=dirpe0xb9q8qiLsFr0=vr0=vr0dc8meaabaqaciaacaGaaeqabaqabeGadaaakeaacqqGhbWrcqqGHbqycqqGWbaCcqqGGaaicqqGwbGvcqqGVbWBcqqGSbaBcqqG1bqDcqqGTbqBcqqGLbqzcqqGGaaicqqGjbqscqqGUbGBcqqGKbazcqqGLbqzcqqG4baEcqGH9aqpdaWcaaqaaiabbEeahjabbggaHjabbchaWjabbccaGiabbAfawjabb+gaVjabbYgaSjabbwha1jabb2gaTjabbwgaLbqaaiabbMeajjabb6gaUjabbsha0jabbwgaLjabbkhaYjabbAgaMjabbggaHjabbogaJjabbwgaLjabbccaGiabbgeabjabbkhaYjabbwgaLjabbggaHbaaaaa@601E@

The smaller the gap volume index, the more complementary the interface shapes are. Gap volume was computed using the SURFNET program [38]. The minimum and maximum radius for gap spheres were set to 1.0 and 5.0 Å, respectively. The grid separation was set to 2.0 Å.

#### Conservation score of the interface

We calculated the conservation scores for residues in the interface as determined by the ConSurf method [21]. The conservation score of the interface was defined as the average value of conservation scores of all the residues at the protein-protein interface. In a similar way to the area-based amino acid composition, we weighted the conservation score for each residue by its ΔSASA upon the formation of the interaction. The average of these weighted residue conservation scores was used as the area-based conservation score of the interface.

For the purpose of clarity, we introduce a set of abbreviations for these interface properties (Table 2).

### Classification method

We employed a support vector machine [39,40] to classify the three types of interactions. In general, an SVM is a supervised learning algorithm for binary classification of data. For more than two classes of data, multi-class techniques are required. These techniques include "one-against-one" and "one-against-all" approaches [41]. For these purposes, several binary SVM classifiers are constructed and the appropriate class is determined using a majority voting scheme. An alternative approach is a multi-stage classifier that separates data progressively. Here, the classification is performed in several stages, and in each stage one class of data is separated.

**Figure 1:**
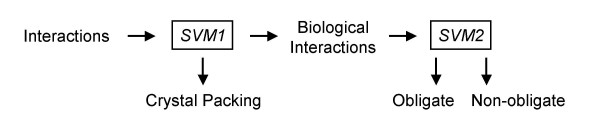
**Schematic plot of the two-stage SVM**. If an interaction is classified as crystal packing by SVM1, it will not be considered by SVM2; otherwise it is classified by SVM2 as either obligate or non-obligate interaction.

We used both a "one-against-one" and a two-stage SVM classifier. In the first stage (SVM1) of the two-stage classification strategy, crystal packing contacts were separated from biological interactions. Then putative biological interactions were passed to the second stage (SVM2), where obligate and non-obligate complexes were distinguished (Figure 1).

The R package *e1071 *[42,43] interfacing to *libsvm *[44] was used to perform the SVM classification. Best results were obtained when radial basis kernels were chosen for SVMs in both stages. To achieve best performance, parameters gamma and C were tuned using the build-in function "tune" in *e1071*. We performed a recursive grid-search for the best parameters using a leave-one-out cross-validation procedure. The parameter search stops when the improvement of accuracy is less than 0.1%. In the best performing two-stage SVM using three interface properties (IA, IAR, and AACa), they were set to 0.004 and 128 for the SVM in the first stage, and 0.00085 and 512 for the SVM in the second stage.

We obtained posterior probabilities for our classification with the same R package. It fits a logistic distribution to the pairwise classification decision values using a maximum likelihood algorithm [44]. With this fitted distribution the posterior pairwise class probabilities are estimated for each prediction.

## Results

### Analysis of interface properties

#### Interface area

**Figure 2:**
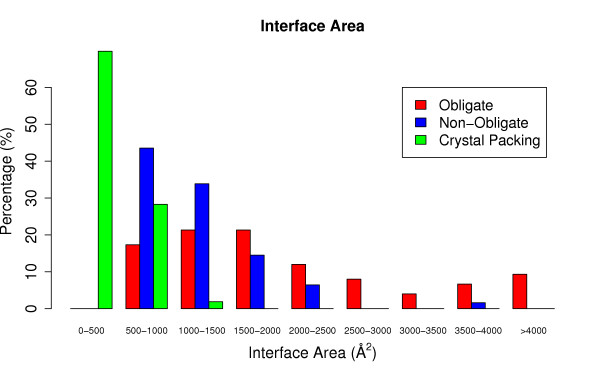
Distribution of interface area for three types of interactions in the BNCP-CS dataset.

The histogram of IAs for the three types of interactions in the BNCP-CS dataset is shown in Figure 2. The average values of IA for obligate, non-obligate and crystal packing interactions are 2156.5 Å^2^, 1170.7 Å^2^, and 435.9 Å^2^, respectively. The distribution of obligate IAs has the largest variance among the three sets. When using a cutoff of 650 Å^2^, approximately 7% of all instances are misclassified in a binary classification discriminating between biological interactions and crystal packing contacts. The three types of interactions exhibit considerable differences regarding this property.

#### Interface area ratio

**Figure 3:**
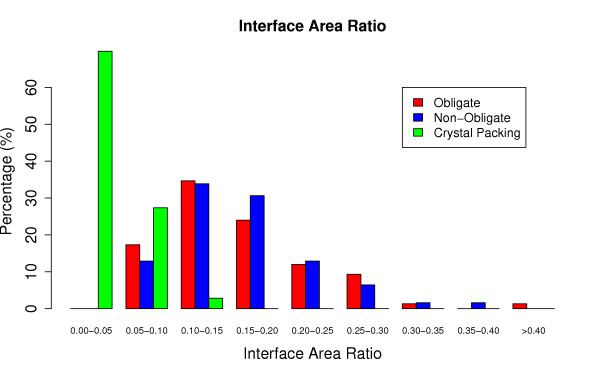
Distribution of interface area ratio for three types of interactions in the BNCP-CS dataset.

The distribution of IARs for the BNCP-CS dataset is shown in Figure 3. The average values of IAR for obligate, non-obligate and crystal packing interactions are 0.16, 0.17, and 0.05, respectively. Using a cutoff of 0.07, approximately 7% of interactions are misclassified in a binary classification discriminating between biological interactions and crystal packing contacts. While the distributions of obligate and non-obligate interactions are similar, both are considerably different from the distribution of the crystal packing contacts.

#### Amino acid composition of the interface

**Figure 4:**
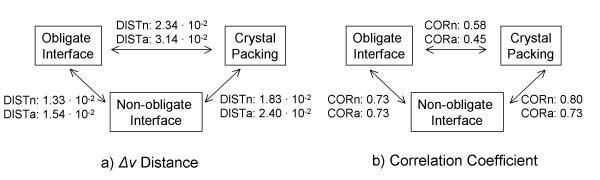
Both Δ*ν *distances (a) and correlation coefficients (b) are shown for each pair of interaction types.

The difference between the AACs of the three types of interactions have been compared in terms of Δ*ν *distances and correlation coefficients (Figure 4). Both AACa and AACn have been used. The lower correlation values and the larger Δ*ν *distance values of area-based composition indicate that area-based composition is a better discriminant than number-based composition for differentiating between the three types of interactions in our study.

**Figure 5:**
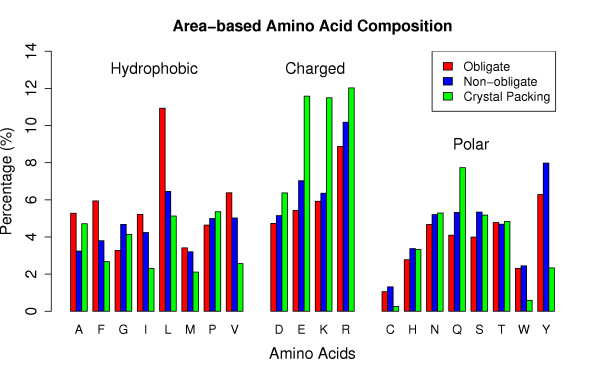
Area-based Amino Acid Composition for three types of interactions in the BNCP-CS dataset.

The overall area-based amino acid composition of the interfaces for the three types of complexes in the BNCP-CS dataset is reported in Figure 5. Hydrophobic residues (FILV) contribute twice as much area to obligate interfaces as to crystal packing contacts. For instance, on average each of the amino acid leucine contributes 46.1 Å^2 ^and 39.5 Å^2 ^to the interface area in obligate and non-obligate interactions, respectively. In contrast, in crystal packing interfaces leucine contributes only around 25.9 Å^2 ^to the interface area. Charged residues (EKR) also show different distributions in the obligate and crystal packing interfaces. Aromatic residues (FWY) tend to be more abundant in biological interfaces. We observed that Cysteine occurs more often in the biological interfaces than in crystal packing contacts. These residues also indicate that non-obligate interfaces exhibit intermediate characteristics between obligate interactions and crystal packing contacts, in particular for the sets of hydrophobic and charged residues.

#### Correlation between amino acid compositions of interface and protein surface

**Figure 6:**
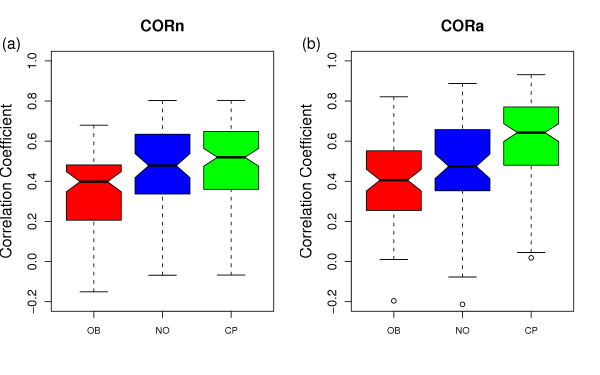
Boxplot of correlation coefficients between amino acid compositions of interface and protein surface for the BNCP-CS dataset, calculated using number-based composition (a) and area-based composition (b).

Correlation coefficients calculated using both number-based and area-based amino acid compositions are reported in Figure 6. The average correlation coefficients for obligate, non-obligate and crystal packing interactions from the BNCP-CS dataset are 0.35, 0.47, and 0.49, respectively, using number-based composition. These average values are 0.39, 0.48, and 0.59 when using area-based composition. Again, non-obligate interactions exhibit intermediate characteristics. The discrimination is more pronounced for area-based correlation.

#### Gap volume index

**Figure 7:**
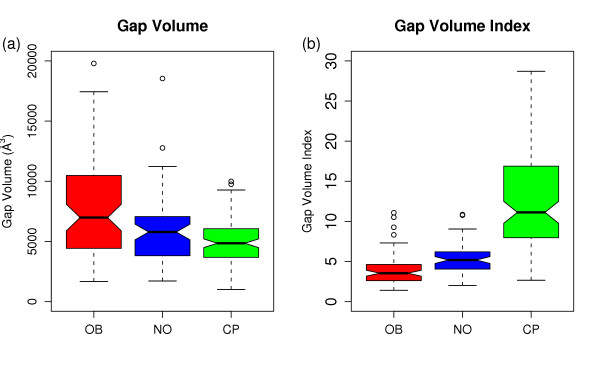
Boxplot of gap volumes (a) and gap volume indices (b) of the BNCP-CS dataset.

It is shown in Figure 7a that obligate and non-obligate interactions tend to have larger gap volumes with respect to the definition for gap used in the SURFNET program. The shape complementarity of the interfaces are indicated by the gap volume index. With regard to gap volume index, obligate and non-obligate interactions have much smaller values than crystal packing contacts (Figure 7b). On average, the gap volume indices are 4.0, 5.3, and 13.8 for obligate, non-obligate interactions, and crystal packing contacts, respectively. Gap volume index discriminates better the three kinds of interactions than gap volume.

#### Conservation score of the interface

**Figure 8:**
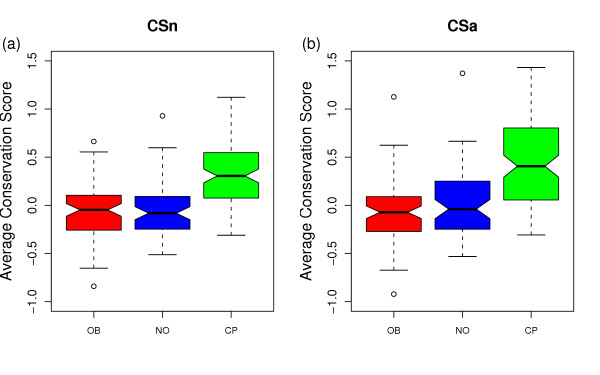
**Boxplot of conservation scores of the interfaces for the   BNCP-CS dataset, calculated using number-based strategy (a),   and area-based strategy (b).  **Lower conservation scores indicate higher degree of conservation.

Figure 8 illustrates that interface residues in obligate and non-obligate interactions are more highly conserved than those in crystal packing contacts. Average area-based conservation scores for obligate and non-obligate interfaces are -0.07 and 0.02, respectively. In contrast, the average area-based conservation score for crystal packing interfaces is 0.44. These results agree with previous observations that interface residues in biological interactions are conserved more strongly [19-22].

**Figure 9:**
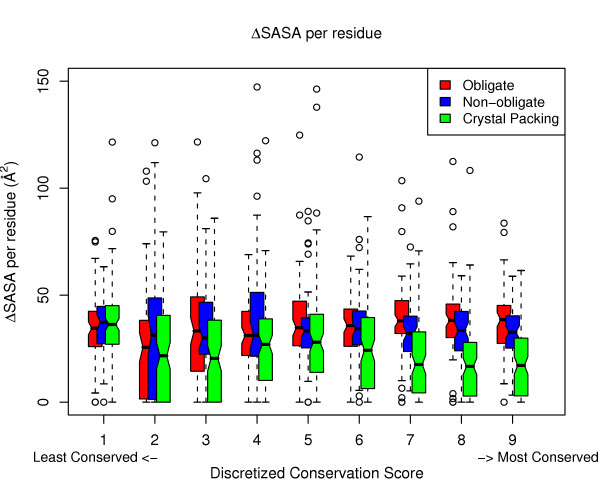
**Average ΔSASA per residue for different degrees of conservation**. Conservation scores from ConSurf are discretized using the same coloring scheme as that used in [48]. The larger the discretized ConSurf scores, the more conserved the residues in evolution. The conserved residues tend to be more strongly involved in the biological interfaces.

In Figure 9, conserved residues in biological interfaces are shown to be more involved in the formation of protein interfaces (high ΔSASA) than those in crystal packing contact with the same degree of conservation. The effect is more pronounced with increasing degree of conservation. On average, ΔSASA for most conserved residues (discretized conservation score equals 9) is 37.6 Å^2 ^and 32.6 Å^2 ^for obligate and non-obligate interactions, respectively, but for crystal packing contacts this value is only 18.6 Å^2^.

#### Relationship between interface properties

Scatter plots comparing different interface properties are provided in the supplementary material (see Additional file 1: supplementary.pdf). In the scatter plots, one can observe that the crystal packing contacts are more clearly separable from the ensemble than the other two types of interactions.

### Performance of the SVM classifiers

#### Leave-one-out cross-validation

We performed leave-one-out cross-validation for the multi-class and two-stage SVMs using the six properties available for the BNCP-CS dataset as input features: IA, IAR, AACa, CORa, GVI, and CSa.

#### Performance measures

The notions true positive (TP), false negative (FN), false positive (FP) and true negative (TN) are defined in Table 3. We used the following performance measures:

Precision=TPTP+FP
 MathType@MTEF@5@5@+=feaafiart1ev1aaatCvAUfKttLearuWrP9MDH5MBPbIqV92AaeXatLxBI9gBaebbnrfifHhDYfgasaacH8akY=wiFfYdH8Gipec8Eeeu0xXdbba9frFj0=OqFfea0dXdd9vqai=hGuQ8kuc9pgc9s8qqaq=dirpe0xb9q8qiLsFr0=vr0=vr0dc8meaabaqaciaacaGaaeqabaqabeGadaaakeaacqqGqbaucqqGYbGCcqqGLbqzcqqGJbWycqqGPbqAcqqGZbWCcqqGPbqAcqqGVbWBcqqGUbGBcqGH9aqpdaWcaaqaaiabdsfaujabdcfaqbqaaiabdsfaujabdcfaqjabgUcaRiabdAeagjabdcfaqbaaaaa@41AD@

Sensitivity=TPPSpecificity=TNN
 MathType@MTEF@5@5@+=feaafiart1ev1aaatCvAUfKttLearuWrP9MDH5MBPbIqV92AaeXatLxBI9gBaebbnrfifHhDYfgasaacH8akY=wiFfYdH8Gipec8Eeeu0xXdbba9frFj0=OqFfea0dXdd9vqai=hGuQ8kuc9pgc9s8qqaq=dirpe0xb9q8qiLsFr0=vr0=vr0dc8meaabaqaciaacaGaaeqabaqabeGadaaakeaafaqabeqacaaabaGaee4uamLaeeyzauMaeeOBa4Maee4CamNaeeyAaKMaeeiDaqNaeeyAaKMaeeODayNaeeyAaKMaeeiDaqNaeeyEaKNaeyypa0ZaaSaaaeaacqWGubavcqWGqbauaeaacqWGqbauaaaabaGaee4uamLaeeiCaaNaeeyzauMaee4yamMaeeyAaKMaeeOzayMaeeyAaKMaee4yamMaeeyAaKMaeeiDaqNaeeyEaKNaeyypa0ZaaSaaaeaacqWGubavcqWGobGtaeaacqWGobGtaaaaaaaa@53CB@

and

Accuracy=Sum of correct predictionsSum of total predictions
 MathType@MTEF@5@5@+=feaafiart1ev1aaatCvAUfKttLearuWrP9MDH5MBPbIqV92AaeXatLxBI9gBaebbnrfifHhDYfgasaacH8akY=wiFfYdH8Gipec8Eeeu0xXdbba9frFj0=OqFfea0dXdd9vqai=hGuQ8kuc9pgc9s8qqaq=dirpe0xb9q8qiLsFr0=vr0=vr0dc8meaabaqaciaacaGaaeqabaqabeGadaaakeaacqqGbbqqcqqGJbWycqqGJbWycqqG1bqDcqqGYbGCcqqGHbqycqqGJbWycqqG5bqEcqGH9aqpdaWcaaqaaiabdofatjabdwha1jabd2gaTjaaykW7cqWGVbWBcqWGMbGzcaaMc8Uaem4yamMaem4Ba8MaemOCaiNaemOCaiNaemyzauMaem4yamMaemiDaqNaaGPaVlabdchaWjabdkhaYjabdwgaLjabdsgaKjabdMgaPjabdogaJjabdsha0jabdMgaPjabd+gaVjabd6gaUjabdohaZbqaaiabdofatjabdwha1jabd2gaTjaaykW7cqWGVbWBcqWGMbGzcaaMc8UaemiDaqNaem4Ba8MaemiDaqNaemyyaeMaemiBaWMaaGPaVlabdchaWjabdkhaYjabdwgaLjabdsgaKjabdMgaPjabdogaJjabdsha0jabdMgaPjabd+gaVjabd6gaUjabdohaZbaaaaa@7E02@

#### Feature selection

We investigated the best performances of the two-stage SVM in terms of cross-validation accuracy when using combinations of six individual features: IA, IAR, AACa, CORa, GVI, and CSa (see Additional file 1: supplementary.pdf). For the BNCP-CS dataset, the best single feature is IA with an accuracy of 76.5%. The best combination of two features is IA and AACa, yielding 86.0%. Using the three features IA, IAR, and AACa, yields 91.8%. With the four features, IA, IAR, AACa, and GVI (or CSa), we obtained 91.4%. The best accuracy is 90.5% when using five features with IA, IAR, AACa, GVI, and CSa. When using all six features the accuracy is 89.7%.

#### Multi-class SVM

The accuracy of the multi-class SVM classifier is slightly below that of the two-stage SVM classifier. With a leave-one-out cross-validation procedure we obtained a best accuracy of 90.9% when using four properties, IA, IAR, AACa, and GVI on the BNCP-CS dataset.

#### Two-stage SVM

Table 4 and Table 5 list the leave-one-out cross-validation results and performances of the two-stage SVM classifiers for the BNCP-CS datasets using three feature combination with highest accuracy (IA, IAR, AACa). The classifier identified crystal packing contacts more accurately than it did for the other two types of interactions. The performance for non-obligate interactions is slightly lower than that for obligate interactions. In total, the accuracy is 91.8% (= 223/243) for the two-stage SVM classifiers. The two stages SVM1 and SVM2, as depicted in Figure 1, have leave-one-out cross-validation accuracies 97.9% and 86.4%, respectively for the BNCP-CS dataset.

#### Test for overfitting with nested cross-validation

By selecting parameters for the SVMs after cross-validation, we followed a standard procedure applied when limited data are available. Ideally, the data should be split into training, parameter optimization, and validation sets. Since our dataset is of limited size, we maximized the size of the training dataset to get the best-performing SVM classifiers. The drawback is that the accuracy estimates are possibly too optimistic. In order to test for overfitting, we estimated the misclassification rate following a previously described nested cross-validation protocol [45]. We divided the data into three parts, on two parts 10-fold cross-validation was performed to train the model and select optimal parameters. On the third part the model was tested. Repeating the whole procedure five times, the average accuracies and standard deviations are 81.4 ± 1.46% (BNCP-CS, multi-class, four features IA, IAR, AACa, and GVI), 83.1 ± 1.16% (BNCP-CS, two-stage, three features IA, IAR, and AACa). For the two-stage SVM, the accuracies for the first and second stage are 94.5 ± 0.92% and 75.2 ± 2.52%, respectively. There is no considerable difference between the two average accuracy values for the best performing multi-class and two-stage SVMs. The low standard deviations indicate that the method is quite robust. Because of the small size of the training dataset, the accuracy estimates from the nested cross-validation might be overly pessimistic.

#### Testing on Bahadur's dataset

We have applied our best performing SVM, which is the two-stage SVM trained using three features (IA, IAR, and AACa), to the dataset used by Bahadur *et al*. [9]. This dataset includes 188 crystal packing contacts, 122 homodimers, and 70 other protein-protein complexes. This dataset has some overlap with the BNCP-CS dataset. Between the two sets there are 36 homodimers and 19 other biological complexes with more than 40% sequence identity. In total, the accuracy of the first stage SVM is 80.0%, which is considerably less than the performance of the first stage SVM on the nested cross validation (94.5 ± 0.92%). This can be explained by the fact that the crystal packing dataset used by Bahadur *et al*. is heavily biased toward crystal packing contacts with large contacting area (> 400 Å^2^).

We can reasonably expect that in this dataset the subset of homodimers mostly includes obligate interactions. In addition, inspecting the descriptions of the 70 other protein-protein complexes in the PDB files, one can expect that this subset mostly contains non-obligate interactions. The second stage SVM predicts 84.4% of the homodimers to be obligate, and 78.6% of the remaining complexes to be non-obligate. Although these results do not represent an actual validation, they do agree with our expectations.

## Discussion

In this paper we analyzed five interface properties for three types of protein-protein interactions. Interface area remains one of the most important features for distinguishing biological interactions from crystal packing contacts. The area of a crystal packing interface is typically smaller than that of a biological interface (Figure 2) Different cutoffs have been proposed for separating crystal packing contacts from biological interactions [5,6]. In our analysis we found 650 Å^2 ^to be a reasonable cutoff of interface area for the binary classification of biological and non-biological interactions. This threshold separates the BNCP-CS dataset with an accuracy of 93%. Biological interactions where small protomers are involved are better identified using the interface area ratio property in addition.

The 20 amino acids display variable preference for protein-protein interaction in terms of the number of residues taking part in the interaction and the ΔSASA involved in the total interface area. Obligate and non-obligate interactions show noticeable differences regarding the features based on amino acid composition.

Residues involved in biological interactions were shown to be more strongly conserved than residues involved in crystal packing contacts (Figure 8). With the increase of conservation scores of the interface residues, the difference between the three types of interactions are more obvious in terms of their ΔSASA per residue. In particular, conserved residues involved in crystal packing contacts tend to have lower ΔSASA values (Figure 9). The SVM classifier did not benefit from including conservation scores. We investigated whether confidence measures for the conservation score improve performance. To this end, we tested the number of sequences used to calculated the ConSurf score as well as the DOPS score [46]. Improvement was only observed when the number of sequences was combined with the conservation score feature in comparison to only using the ConSurf score as a single feature (55% to 60% improvement using multi-class SVM). No significant improvement was observed when using the number of sequences in addition to the five other features. The effect of confidence measures and conservation scores in the SVM performance deserve further investigation.

As demonstrated in the section on the analysis of the interface properties, the non-obligate interactions in our datasets exhibit intermediate values for all interface properties except the interface area ratio. These results agree with the expected different stability of these types of interactions [10]. Recently, Gunasekaran and coworkers examined the structural properties of ordered and disordered proteins [27]. According to their description, ordered proteins are involved in either non-obligate interactions or crystal packing contacts, while disordered proteins are involved in obligate interactions. The authors have shown that ordered proteins have significantly smaller per-residue SASA at both interface and surface than disordered proteins. These results are in agreement with our analysis. In addition, protomers involved in non-obligate interactions are shown to resemble the protomers involved in crystal packing contacts. Recently, De *et al*. published the results of a statistical analysis of the interface properties for obligate and non-obligate interactions [28]. Our conclusions agree with their results with respect to the interface properties of interface area, residue propensities at the interface, and shape complementarity.

The first stage of the two-stage SVM classifier distinguishes crystal packing contacts from biological interactions with an accuracy of 97.9% (see the Two-stage SVM section). Valdar and Thornton obtained an accuracy of 98.3% on a similar problem [12]. Nevertheless, the performances of the two methods are not directly comparable because the datasets are different and, in particular, the biological interactions were restricted to homodimers in the latter method.

The nested cross-validation results indicate that there is no considerable difference between the performances of the multi-class and two-stage SVMs. The small variances of these results along with the minor difference between the performances of the SVM implementations indicate that the approach is quite robust.

The method based on atomic contact vectors described by Mintseris and Weng results in considerable accuracy (91%) in the classification of obligate and non-obligate interactions [30]. We intend to integrate this type of feature in a future version of NOXclass.

This study is also related to the work of Bradford and Westhead, investigating different interaction types [25]. However, the aims of the two studies are different. Bradford and Westhead identify the possible binding site at the surface of a given protein, while we use the structural model of the complex to determine the interaction types. Although the oligomeric states of many proteins may be inferred during the process of protein purification for crystallization, this is not always the case. In addition, this information is not easily available in the literature or well annotate in structural databases like the Protein Data Bank (PDB). There is a current lack of a well-defined criterion for defining interaction types based on experimental results, but there has been some recent progress in this area [26].

## Conclusion

In this work we have analyzed several interface properties for three types of protein-protein interactions, *i.e*. obligate interactions, non-obligate interactions, and crystal packing contacts. These three types of interactions exhibit distinct interface properties.

To classify the three types of interactions, we have combined the properties using a support vector machine algorithm and implemented it as NOXclass. NOXclass allows the interpretation and analysis of protein quaternary structures. In particular, it generates testable hypothesis regarding the nature of protein-protein interactions, when experimental results are not available. We can expect this server will benefit the users of protein structural models, as well as protein crystallographers and NMR spectroscopists.

## Availability and requirements

### Program home page

A web server based on the method and the datasets used in this study are available at [47]. Source code for the program can be downloaded from the same address.

### System requirement

NOXclass requires LINUX or UNIX operation system, as well as a Python interpreter.

### External program requirement

The NOXclass program uses NACCESS [35] to calculate the solvent accessible surface areas for residues. The LIBSVM [44] package is required by NOXclass to operate. These two programs are not distributed in the NOXclass package and the users must obtain these programs by themselves for executing the NOXclass program on their local computer.

In addition, the NOXclass program uses SURFNET [38] to compute the gap volume between two protomers. Users have to obtain this program for including this feature in the prediction. Similarly, to include evolutionary information in the prediction, the users must obtain the corresponding conservation scores for their protein sequences from the ConSurf server [21].

### License

The source code of the NOXclass program is distributed under the terms of GNU LGPL.

## List of abbreviations

A list of abbreviations used in this paper has been given in table 2.

## Authors' contributions

HZ developed the method under the supervision of FD, IS and TL. HZ, FD and IS evaluated and interpreted the results. Every author contributed to the final version of the paper.

## Supplementary Material

Additional File 1 supplementary Click here for file

## Figures and Tables

**Table 1:** Dataset BNCP-CS^a^

Obligate Interactions (75)									
1ahj A B	1b34 A B	1dce A B	lefv A B	1gux A B	1h2a L S	1luc A B	1pnk A B		
1req A B	1tco A B	2aai A B	1aOf A B	1a4i A B	1afw A B	1aj8 A B	1ajs A B		
1aom A B	1aq6 A B	1at3 A B	1b3a A B	1b5e A B	1b7b A C	1b8a A B	1b8j A B		
1b9m A B	1bjn A B	1bol A B	1brm A B	1byf A B	1byk A B	1c7n A B	1cli A B		
1cmb A B	1cnz A B	1coz A B	1cp2 A B	1dor A B	1f6y A B	1gpe A B	1hgx A B		
1hjr A C	1hss A B	1isa A B	1jkm A B	1kpe A B	1msp A B	1nse A B	1one A B		
1pp2 L R	1qae A B	1qax A B	1qbi A B	1qfe A B	1qfh A B	1qor A B	1qu7 A B		
1smt A B	1sox A B	1spu A B	1trk A B	1vlt A B	1vok A B	1wgj A B	1xik A B		
1xso A B	1ypi A B	1yve I J	2ae2 A B	2hdh A B	2hhm A B	2nac A B	2pfl A B		
2utg A B	3tmk A B	4mdh A B							

Non-obligate Interactions (62)									

1ava A C	1avw A B	1bvn T P	1cse I E	1eai C A	1f34 A B	1fss A B	1gla F G		
1kxq H A	1smp I A	1tab I E	1tgs I Z	2ptc I E	2sic I E	4sgb I E	1agr E A		
1atn A D	1b6c A B	1bkd R S	1buh A B	1dow A B	1euv A B	1i2m A B	1i8l A C		
1kac A B	1pdk A B	1qav A B	1tx4 A B	1cOf S A	1zbd A B	1ak4 A D	1d09 A B		
1cqi A B	1fin A B	1dhk A B	1bi7 A B	1wql R G	1rrp A B	1ccO A E	1eg9 A B		
1avz B C	1frv A B	3hhr A B	1ycs A B	1cvs A C	1aro L P	1cmx A B	1bml A C		
2pcb A B	1fGO A B	1stf E I	1emv A B	1uea A B	1qbk B C	1hlu A P	1itb A B		
1eth A B	1jtd A B	1lfd A B	1dnl A B	1tmq A B	1a4y A B				

Crystal Packing Contacts (106)									

1k55	1ual	1mxr	1j98	1e9g	1iup	1is3	1gy7	1jzl	1jke
1km1	1ihr	2btc	1eq9	1qf8	1k8u	1m7g	1p5z	1e19	1k75
1iat	1m9f	1ht9	1hqs	1b8z	1lc5	1gs5	1gve	1k20	1i4u
1k9u	1e58	1es9	1qkm	1j8b	1kli	1eyv	1j24	1h1y	1ijy
1exq	1lw6	1m7y	1n31	1nms	1peO	1f6b	1jP3	1kqp	1j79
1mxi	1my7	1k4i	1jat	1f1m	1jdO	1nrv	1mvo	1m2d	1f7z
1gyo	1fs8	1b67	1kzk	1nxm	1k94	1iOr	1euv	1qlO	1g2y
1mh9	1ed9	1dtd	1ld8	1jlt	1ct4	1nsz	1iq6	1i2m	llqp
1lqv	1n2e	1i12	1ubk	1g8q	1e87	1jl0	1jr8	1qip	1nf9
1g60	1uaq	1ozu	1dmh	1eye	1i52	1fjj	1b16	1e4m	31yn
1ock	1icr	1iOd	1jtg	1elu	1kic				

^a^One PDB entry can contain several interfaces of different types. Therefore the same PDB entry can appear in different subsets. For example, 1i2m has a non-obligate interaction between chains A and B. At the same time, the contact between chains B and D is included under the crystal packing contact subset.

**Table 2:** List of Interface Properties

AACa	Amino Acid Composition of the interface, Area-based
AACn	Amino Acid Composition of the interface, Number-based
CORa	CORrelation between amino acid compositions of interface and surface, Area-based
CORn	CORrelation between amino acid compositions of interface and surface, Number-based
CSa	Conservation Score of the interface, Area-based
CSn	Conservation Score of the interface, Number-based
DISTn	Δ*ν *DISTance between amino acid compositions of the interfaces, Number-based
DISTa	Δ*ν *DISTance between amino acid compositions of the interfaces, Area-based
GV	Gap Volume
GVI	Gap Volume Index
IA	Interface Area
IAR	Interface Area Ratio
SASA	Solvent Accessible Surface Area

**Table 3:** Definitions of Notions TP, FN, FP, and TN

		**Predicted**		**Total**
		**Type X^a^**	**¬Type X**	
**True**	**Type X**	TP	FN	P
	**¬Type X**	FP	TN	N

^a^Type X can be any of the three types of interactions.

**Table 4:** Leave-one-out cross-validation results for the BNCP-CS dataset using the two-stage SVM^a^

		**Predicted**			**Total**
		**OB^b^**	**NO^c^**	**CP^d^**	
	**OB**	69	6	0	75
**True**	**NO**	9	52	1	62
	**CP**	3	1	102	106

**Total**		81	59	103	243

^a^Three out of the six properties (IA, IAR, and AACa) are used in the SVM classification for the BNCP-CS dataset;

^b^OB: Obligate interactions;

^c^NO: Non-obligate interactions;

^d^CP: Crystal packing contacts.

**Table 5:** Performance of the two-stage SVM classifier^a^

**Interaction Type**	**OB^b^**	**NO^c^**	**CP^d^**
**Precision**	85.2%	88.1%	99.0%
**Sensitivity**	92.0%	83.9%	96.2%
**Specificity**	96.3%	94.6%	97.1%

**SVM stage**	**Stage 1**	**Stage 2**	**Combined**

**Accuracy**	97.9%	86.4%	91.8%

^a^Three out of the six properties (IA, IAR, and AACa) are used in the SVM classification for the BNCP-CS dataset;

^b^OB: Obligate interactions;

^c^NO: Non-obligate interactions;

^d^CP: Crystal packing contacts.
