# Supplementary material for: NOXclass: prediction of protein-protein interaction types
Source: BMC Bioinformatics. 2006 Jan 19;7:27. doi: 10.1186/1471-2105-7-27 (PMC1386716; doi:10.1186/1471-2105-7-27)
Supplement: Additional File 1 — supplementary [file 1471-2105-7-27-S1.pdf]

Table 1: Leave-one-out cross-validation (LOOCV) results

Interface Properties						Multi-class SVM	Two-stage SVM
IA[a]	IAR[b]	AACa[c]	CORa[d]	GVI[e]	CSa[f]	Acc.(%)	Acc.(%)
X						76.1	76.5
	X					67.9	67.9
		X				74.9	74.9
			X			53.1	51.4
				X		72.0	72.0
					X	56.4	32.5
X	X					83.5	84.8
X		X				82.7	86.0
X			X			78.2	78.6
X				X		79.4	79.8
X					X	77.8	77.4
	X	X				79.0	80.7
	X		X			72.8	72.4
	X			X		81.9	82.3
	X				X	71.2	72.4
		X	X			74.9	77.0
		X		X		76.1	77.4
		X			X	76.5	77.4
			X	X		68.7	70.0
			X		X	62.1	62.1
				X	X	74.9	76.1
X	X	X				90.1	**91.8**
X	X		X			84.8	87.7
X	X			X		87.7	89.3
X	X				X	83.5	84.0
X		X	X			84.4	85.2
X		X		X		82.3	84.8
X		X			X	84.0	87.7
X			X	X		81.5	83.5
X			X		X	79.8	81.1
X				X	X	79.4	83.5
	X	X	X			79.4	81.9
	X	X		X		78.6	81.1
	X	X			X	79.4	80.7
	X		X	X		79.4	81.1
	X		X		X	73.3	74.5
	X			X	X	83.5	81.9
		X	X	X		77.0	78.2
		X	X		X	77.4	79.0
		X		X	X	79.0	81.1
			X	X	X	74.1	74.0
X	X	X	X			89.3	90.9
X	X	X		X		**90.9**	91.4
X	X	X			X	90.5	91.4
X	X		X	X		85.2	89.7
X	X		X		X	86.8	87.7
X	X			X	X	86.8	88.9
X		X	X	X		81.1	84.8
X		X	X		X	86.8	87.7
X		X		X	X	85.2	87.2
X			X	X	X	81.9	85.2
	X	X	X	X		79.8	81.9
	X	X	X		X	80.7	81.5
	X	X		X	X	80.2	81.9
	X		X	X	X	80.7	82.7
		X	X	X	X	79.8	82.3
X	X	X	X	X		87.7	90.1
X	X	X	X		X	89.7	89.3
X	X	X		X	X	88.1	90.5
X	X		X	X	X	88.1	89.3
X		X	X	X	X	85.6	86.4
	X	X	X	X	X	80.2	83.1
X	X	X	X	X	X	88.5	89.7

[a]IA: Interface Area;

[b]IAR: Interface Area Ratio;

[c]AACa: Area-based Amino Acid Composition;

[d]CORa: CORrelation between area-based amino acid compositions of interface and protein surface;

[e]GVI: Gap Volume Index;

[f]CSa: Area-based Conservation Score of the interface.

Table 2: Leave-one-out cross-validation results for the BNCP-CS dataset using the multi-class SVM[a]

**Dataset**			**Predicted**			
			**OB**[b]	**NO**[c]	**CP**[d]	**Total**
		**OB**	68	7	0	75
**BNCP-CS**	**True**	**NO**	9	51	2	62
		**CP**	3	1	102	106
		**Total**	80	59	104	243

[a]Four out of the six interface properties have used in the SVM classification for the BNCP-CS dataset: interface area, interface area ratio, area-based amino acid composition, and gap volume index;
[b]OB: Obligate interactions;
[c]NO: Non-obligate interactions;
[d]CP: Crystal packing contacts.

Table 3: Performance of the multi-class SVM[a]

	**OB**[b]	**NO**[c]	**CP**[d]	**Combined**
**Precision**	85.0%	86.4%	98.1%	-
**Sensitivity**	90.7%	82.3%	96.2%	-
**Specificity**	95.7%	94.0%	97.1%	-
**Accuracy**	-	-	-	90.9%

[a]Same properties are used as in Table 2;
[b]OB: Obligate interactions;
[c]NO: Non-obligate interactions;
[d]CP: Crystal packing contacts.

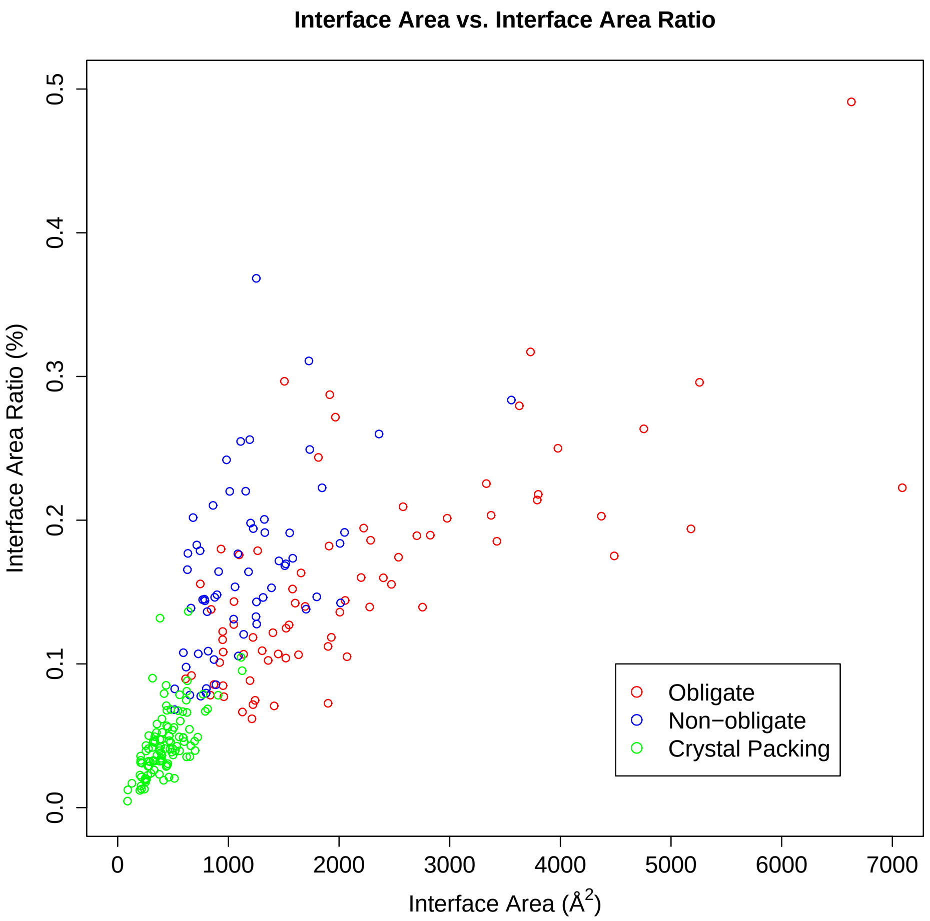

Figure 1: Scatter plot (interface area *vs.* interface area ratio) for interactions in the BNCP-CS dataset.

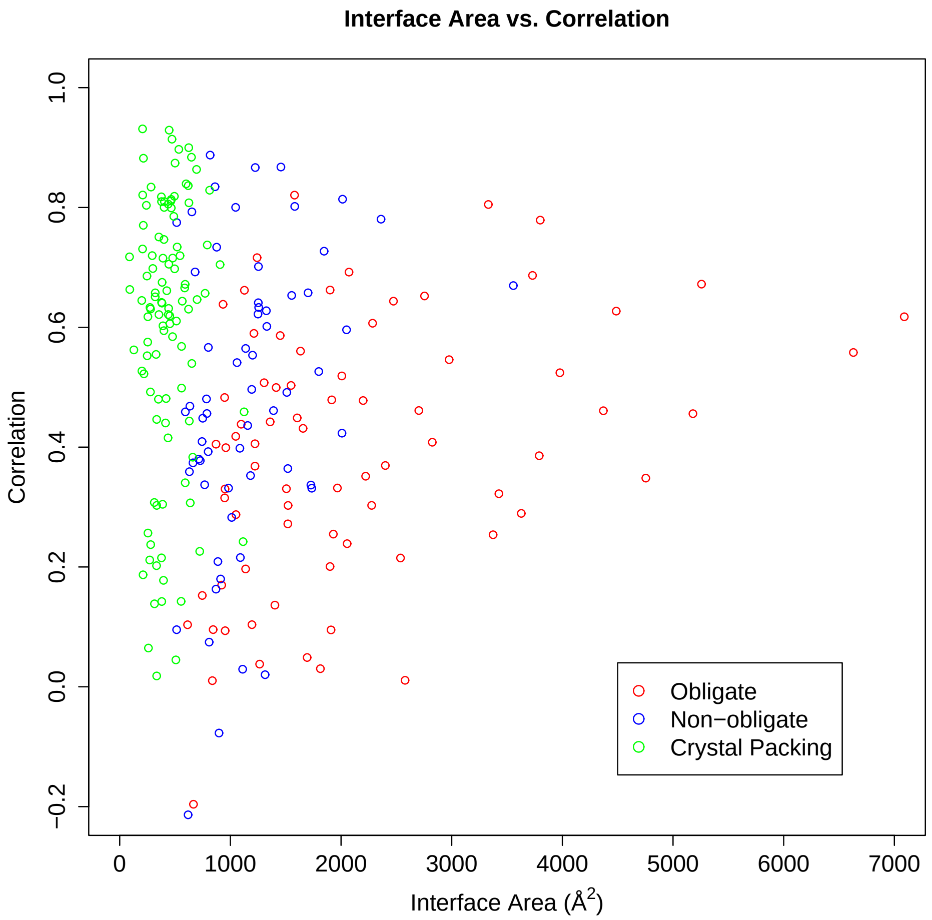

Figure 2: Scatter plot (interface area *vs.* correlation between area-based amino acid compositions of interface and protein surface) for interactions in the BNCP-CS dataset.

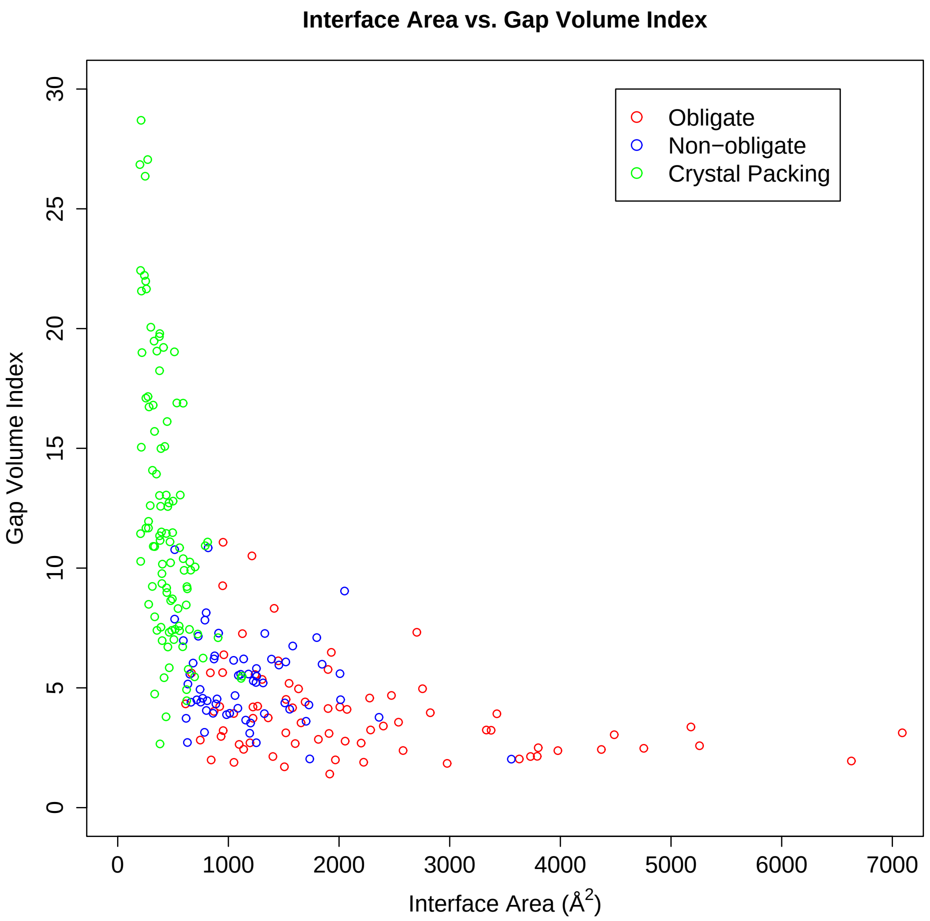

Figure 3: Scatter plot (interface area *vs.* gap volume index) for interactions in the BNCP-CS dataset.

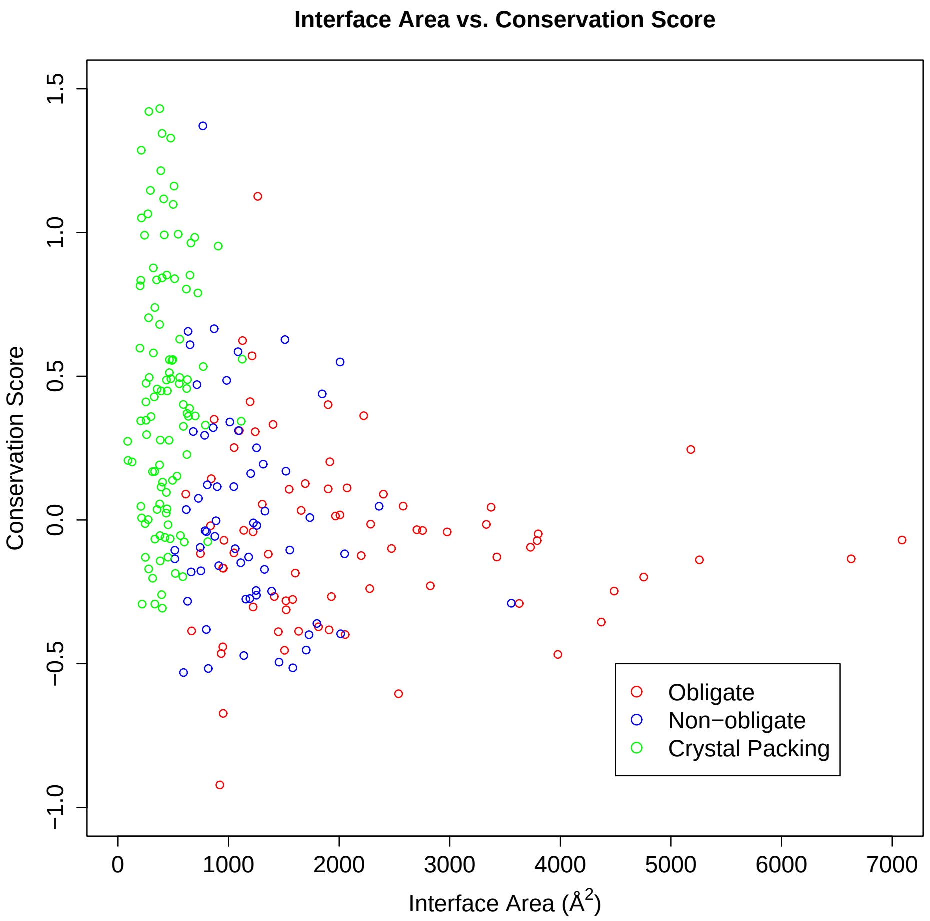

Figure 4: Scatter plot (interface area *vs.* conservation score of the interface) for interactions in the BNCP-CS dataset.

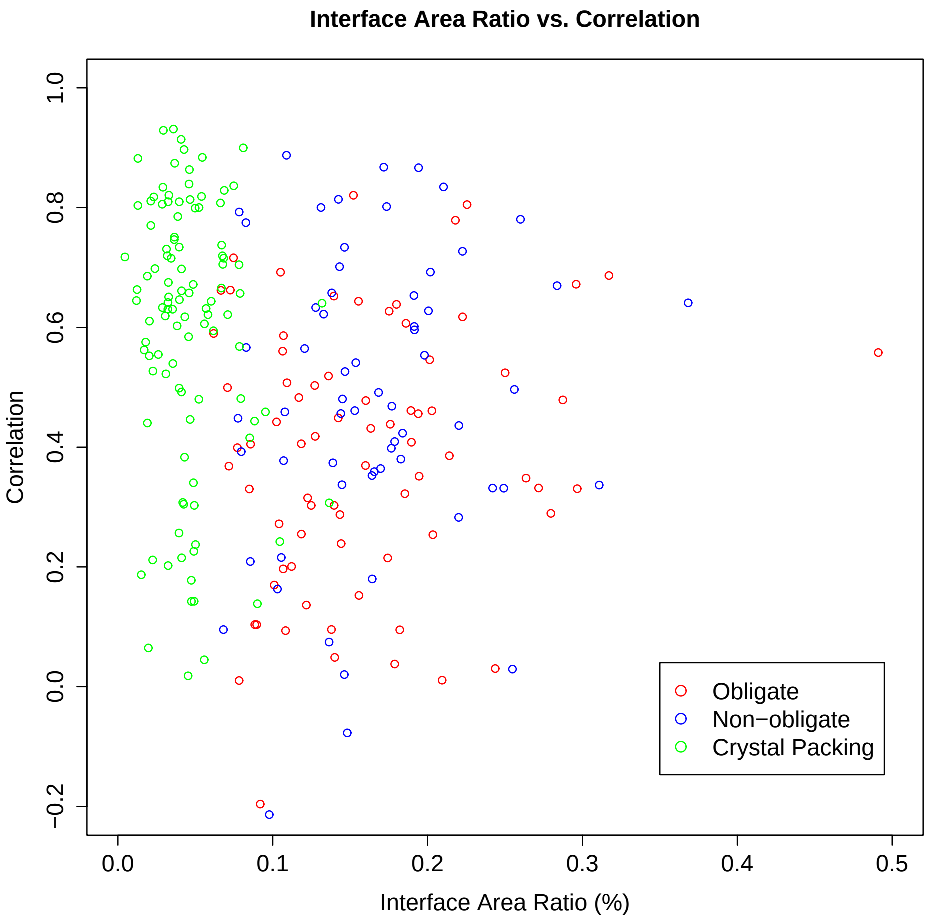

Figure 5: Scatter plot (interface area ratio *vs.* correlation between area-based amino acid compositions of interface and protein surface) for interactions in the BNCP-CS dataset.

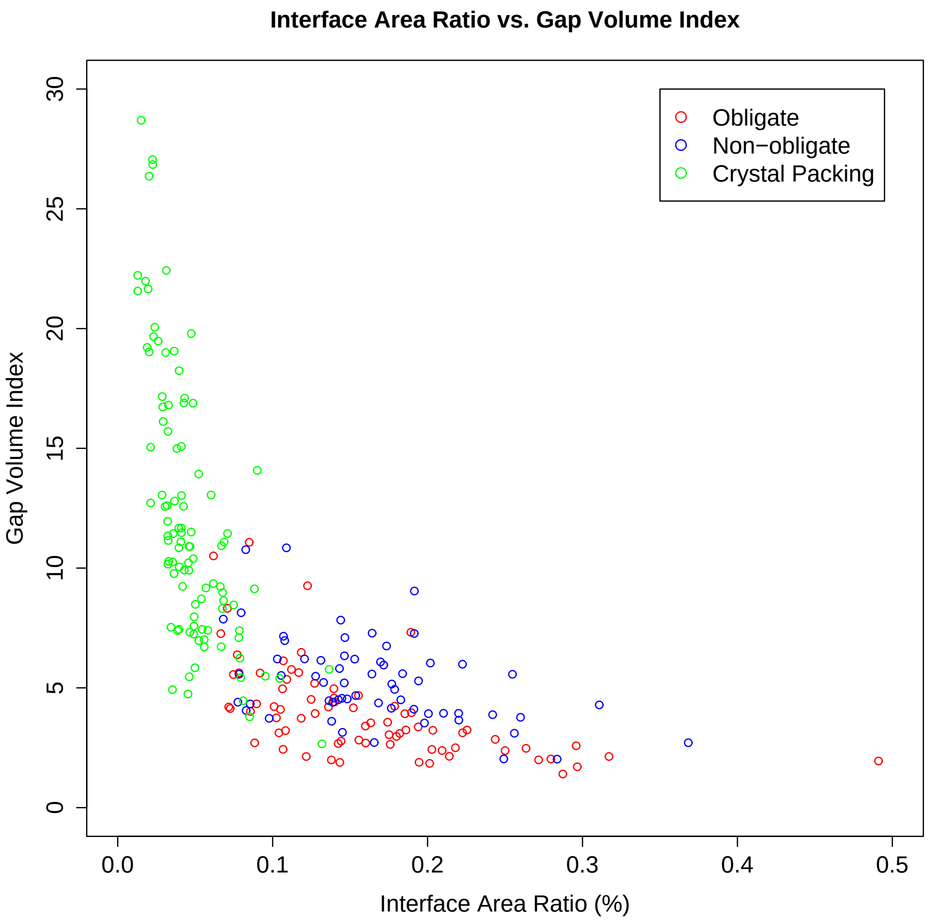

Figure 6: Scatter plot (interface area ratio *vs.* gap volume index) for interactions in the BNCP-CS dataset.

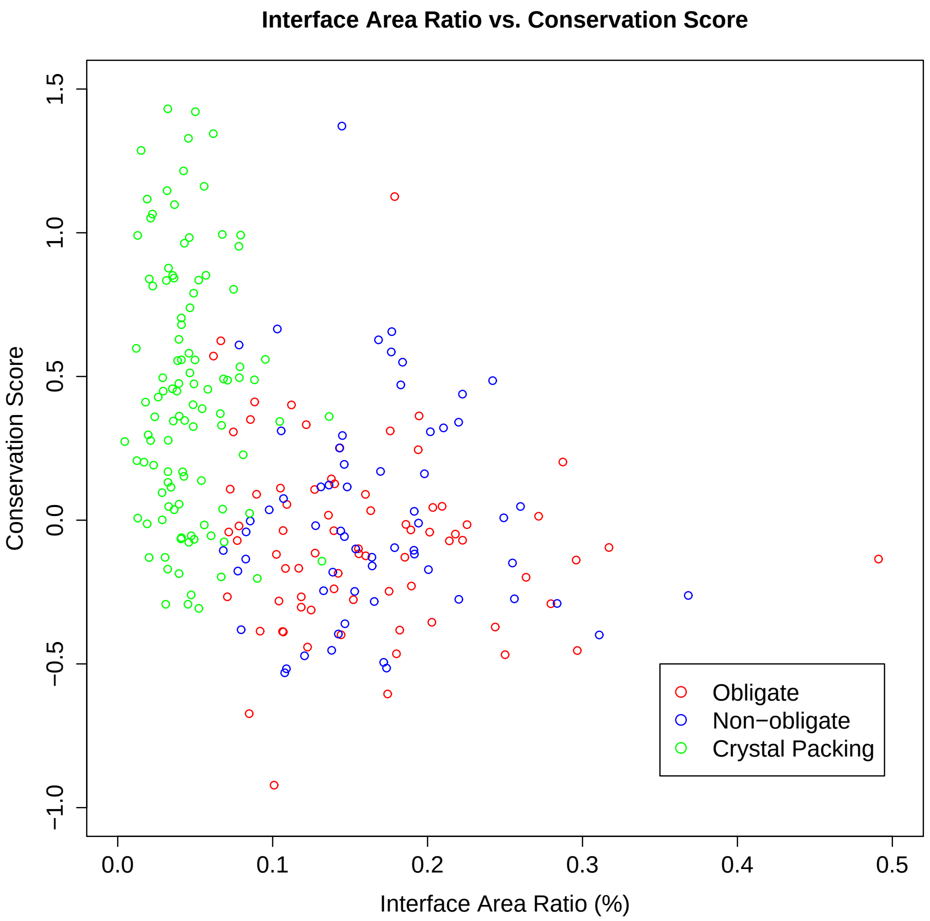

Figure 7: Scatter plot (interface area ratio *vs.* conservation score of the interface) for interactions in the BNCP-CS dataset.

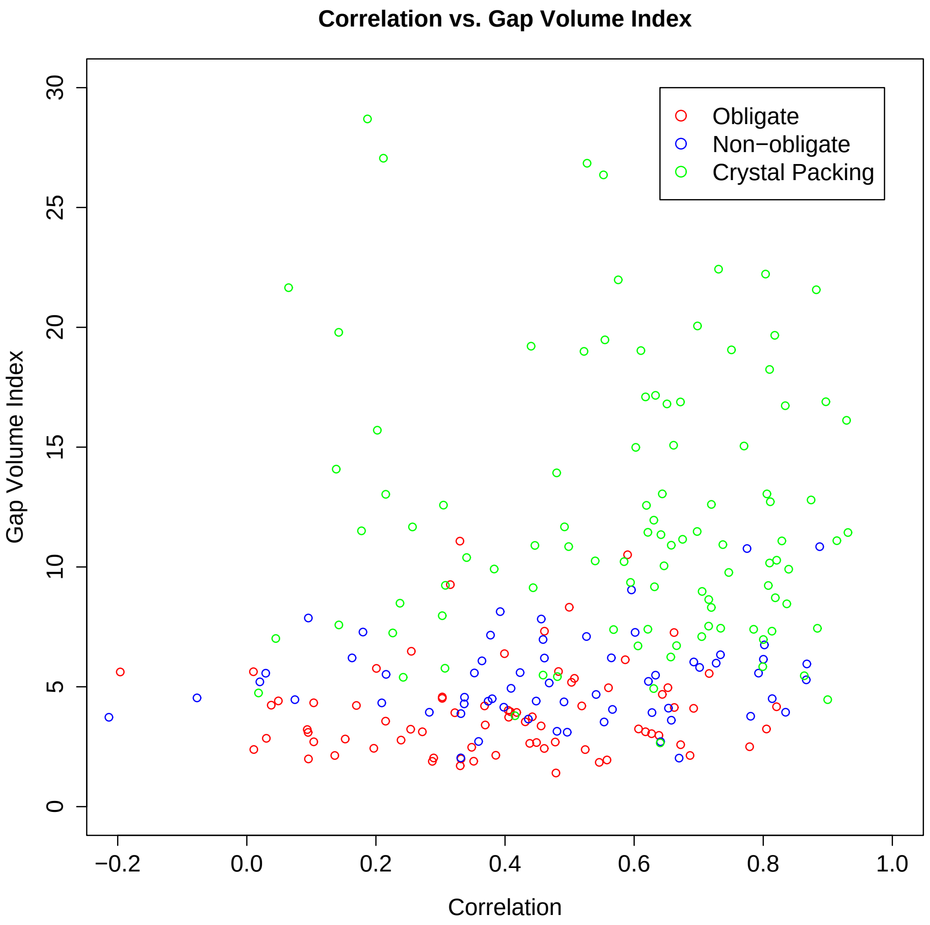

Figure 8: Scatter plot (correlation between area-based amino acid compositions of interface and protein surface *vs.* gap volume index) for interactions in the BNCP-CS dataset.

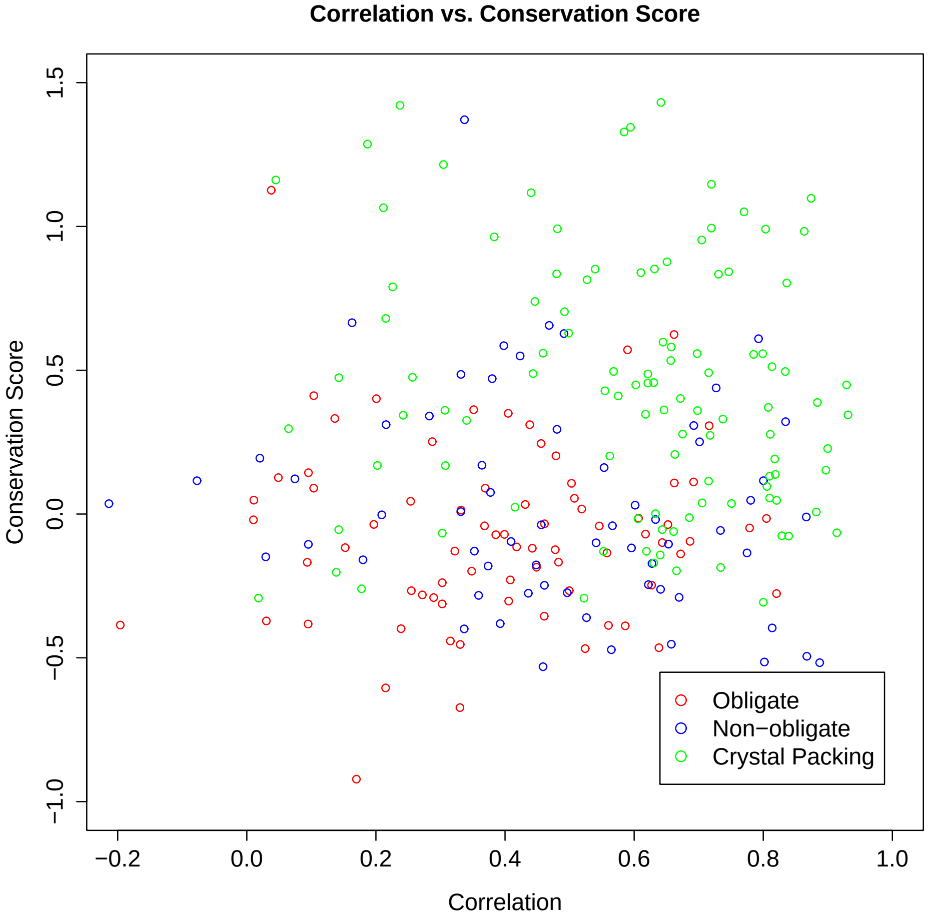

Figure 9: Scatter plot (correlation between area-based amino acid compositions of interface and protein surface *vs.* conservation score of the interface) for interactions in the BNCP-CS dataset.

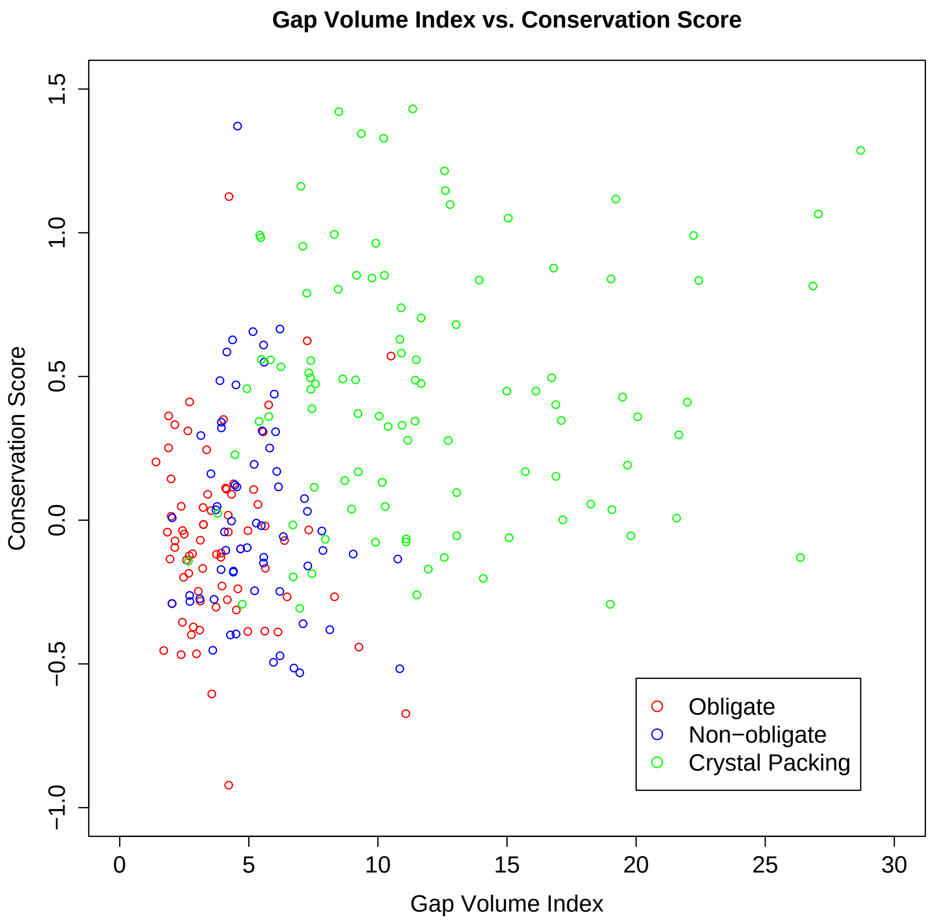

Figure 10: Scatter plot (gap volume index *vs.* conservation score of the interface) for interactions in the BNCP-CS dataset.
